# Intestinal NF-κB and STAT signalling is important for uptake and clearance in a *Drosophila-Herpetomonas* interaction model

**DOI:** 10.1371/journal.pgen.1007931

**Published:** 2019-03-01

**Authors:** Lihui Wang, Megan A. Sloan, Petros Ligoxygakis

**Affiliations:** Laboratory of Cell Biology, Development and Genetics, Department of Biochemistry, University of Oxford, Oxford, United Kingdom; Seoul National University, REPUBLIC OF KOREA

## Abstract

Dipteran insects transmit serious diseases to humans, often in the form of trypanosomatid parasites. To accelerate research in more difficult contexts of dipteran-parasite relationships, we studied the interaction of the model dipteran *Drosophila melanogaster* and its natural trypanosomatid *Herpetomonas muscarum*. Parasite infection reduced fecundity but not lifespan in NF-κB/Relish-deficient flies. Gene expression analysis implicated the two NF-κB pathways Toll and Imd as well as STAT signalling. Tissue specific knock-down of key components of these pathways in enterocytes (ECs) and intestinal stem cells (ISCs) influenced initial numbers, infection dynamics and time of clearance. *Herpetomonas* triggered STAT activation and proliferation of ISCs. Loss of Relish suppressed ISCs, resulting in increased parasite numbers and delayed clearance. Conversely, overexpression of Relish increased ISCs and reduced uptake. Finally, loss of Toll signalling decreased EC numbers and enabled parasite persistence. This network of signalling may represent a general mechanism with which dipteran respond to trypanosomatids.

## Author summary

Neglected Tropical Diseases are the most common diseases of the world’s poorest people. Many are caused by parasites called trypanosomatids that are transmitted to humans via insects belonging to the order of Diptera (also known as true flies). These flies (including tsetse, sand flies and black flies) are difficult to study in the lab and so the prospect of rapid progress in the basic biology of fly-parasite interaction is bleak. However, a model dipteran species with an extensive “tool-box” is the fruit fly *Drosophila melanogaster* with its natural trypanosomatid *Herpetomonas muscarum*. Here we establish the framework of their interaction with the view that part of this interaction will represent an evolutionary conserved component of the dipteran response to parasite infection and will inform more targeted studies into medically important but difficult to study Diptera.

## Introduction

Neglected Tropical Diseases (NTDs) like sleeping sickness, leishmaniasis, hookworm infections, river blindness and elephantiasis are the most common infections of the world’s 1.4 billion poorest people and the leading causes of chronic disability and poverty [1,2]. NTDs are found mostly (but not only see [1]) in low and middle income countries [3]. For NTDs communicated to humans through an insect vector, the ability of the pathogen to overcome the insect’s midgut defenses is absolutely central to transmission. This is clearly illustrated by the following examples.

African trypanosomes, responsible for sleeping sickness and nagana, encounter a severe barrier to their establishment in the midgut of their tsetse fly vectors [reviewed 4]. It has been shown that there is increased resistance to *Trypanosoma brucei spp* (*T*. *brucei spp*) from their first blood meal where 50% of *T*. *brucei spp* become established, to their third blood meal onwards (the fly may take 40–60 blood meals in its life) where less than 10% of challenged flies become infected [5]. Paradoxically therefore, given their importance as vectors, tsetse fly populations are overwhelmingly resistant to trypanosome infection and the resistance mechanisms are manifested largely in the fly midgut [reviewed in 6,7].

Leishmania parasites seem to have successfully overcome barriers to establishment in their sand fly hosts as they develop in large numbers in the midgut of challenged laboratory strains. Nevertheless, in the wild there is only 1% of caught sandflies infected with Leishmania indicating that most infected sandflies are able to clear the parasite [reviewed in 8]. In the case of filariasis, the numbers of microfilaria ingested by all vectors (black flies, mosquitoes etc.) decline dramatically in the midgut lumen with either none or only a small fraction managing to penetrate the midgut barrier. Again, permissiveness of the mosquito midgut for parasite invasion is a key factor in determining success of the infections [9] but we understand little at the molecular level of the mechanisms involved.

In mosquitoes also, establishment of the malaria parasite *Plasmodium falsiparum* is dependent on overcoming midgut defenses [10]. Unlike mosquitoes however, where technological development has been rapid, for some dipteran vectors challenged with kinetoplastid parasites the “tool box” required to tease out these interactions is being developed rather more slowly. For example, there is no realistic prospect of producing transgenic technology for tsetse flies because eggs are inaccessible due to intrauterine development of larvae; there is currently no transgenic technology for sandflies; maintenance of multiple lines of both flies permitting genetic studies is costly and complex; bioinformatics resources are in their infancy. In this context, the model dipteran insect *D*. *melanogaster* may be able to give answers on the possible existence of an evolutionary conserved component of the dipteran host response to kinetoplastid parasites.

Like all insects, *Drosophila* possesses a sophisticated antimicrobial defense. This is rapidly activated upon immune challenge by NF-*κ*B-like transcription factors through two distinct signaling cascades, namely the Toll and IMD pathways. Sensing of *β*-1,3-glucan of fungi and peptidoglycan of Gram-positive bacteria principally trigger Toll signaling. This activation centers on the transmembrane receptor Toll, which is activated by the endogenous ligand Spz, a Nerve Growth Factor homologue [11]. Signal transduction through a receptor-proximal complex including Myd88, Tube and Pelle culminates in the proteolysis of the *Drosophila* IκB Cactus, which enables the translocation to the nucleus of the NF-κB homologue DIF [12]. Moreover, peptidoglycan from Gram-negative bacteria and Gram-positive bacilli primarily induce the IMD pathway, homologous to the TNFR1 pathway. Upon recognition by Peptidoglycan Recognition Proteins PGRP-LC and PGRP-LE, the signal is transmitted to Imd itself (a RIP-1 homologue) and then to TGF-β Activating Kinase 1 (TAK1), which phosphorylates and thus activates, the fly IκB Kinase (IKK) complex [13]. In turn, IKK phospholylates and the caspase-8 homologue Dredd cleaves the composite NF-κB/IκB Relish transcription factor, releasing the N-terminal DNA-binding part of the protein to translocate to the nucleus [14] and regulate hundreds of genes including several antimicrobial peptides (AMPs) [15]. In addition, the *Drosophila Ja*nus *K*inase/*S*ignal *T*ransducer and *A*ctivator of *T*ranscription (JAK-STAT) pathway has been implicated in defenses against viruses as well as a stress-response mechanism utilizing a set of core signaling components [reviewed in 16]. A transmembrane receptor encoded by *domeless* (*dome*), a single JAK tyrosine kinase encoded by *hopscotch* (*hop*), the transcription factor *stat92E* and *unpaired* (*upd*), as well as two related ligands encoded by *upd2* and *upd3*. Binding of Upd ligands to the Dome receptor leads to activation of Hop, which phosphorylates itself and Dome. Cytoplasmic Stat92E can bind to phosphorylated Dome/Hop complexes. Once bound to the Dome/Hop complexes, Stat molecules are phosphorylated and forming Stat dimers that will translocate to the nucleus and regulate target genes [16]. Both Imd and JAK/STAT pathway are involved in systemic as well as in epithelial immunity, especially gut epithelial immunity. There, the *Drosophila* midgut contains pluripotent intestinal stem cells (ISCs) that have a simple lineage: each ISC divides asymmetrically to produce itself and a transient enteroblast (EB), which will undergo terminal differentiation into either an polyploid absorptive enterocyte (EC) or as a diploid secretory enteroendocrine cell (EE) [17,18]. Individual ISCs are scattered along a thin layer of basal lamina in the posterior midgut and are the only proliferating cells in the epithelium [17,18]. This proliferation can be marked with an antibody against the phosphorylated form of Histone-3 (PH3^+^ cells) [19,20].

Very few studies of kinetoplastid interactions with *Drosophila* have been published. One biochemical study has looked at AMP production in response to infection with *Crithidia* spp [21]. In addition to not being a natural parasite for *Drosophila*, *Crithidia* largely infect the rectum of flies and are not a good model for midgut vector–parasite interactions. Nevertheless, natural gut-dwelling kinetoplastid parasites of *Drosophila* do exist [22]. A potential model system of greater relevance is *Herpetomonas ampelophilae*, a natural kinetoplastid parasite of *D*. *melanogaster*, which establishes infection in the midgut of the fly and can go on to invade the salivary gland [23, 24]. However, there are no studies that have examined the interaction between the adult *D*. *melanogaster* midgut and *Herpetomonas* beyond those initial papers. A recent study, has described the interaction between *Drosophila falleni* and *Jaenimonas drosophilidae*, a novel natural trypanosomatid parasite isolated from the wild [25]. There, *D*. *falleni* larvae were persistently infected throughout development, demonstrating persistent infection. However, there was a pronounced bottleneck in infection over metamorphosis with substantially lower rates of Infection in adults than in larvae (Hamilton *et al*, 2015). Using the rate of initial infectivity as a measure, these authors showed that *J*. *drosophilidae* infection in *D*. *melanogaster* larvae provoked an immune response that was not dependent on the IMD pathway [25].

In the present work we have developed a *Drosophila-Herpetomonas* system to be able to dissect insect-parasite interactions in a model dipteran insect. Using transcriptomics as well as tissue-specific RNAi assays, we have pinpointed the activation of the immune pathways responsible for gut defences upon parasite infection and documented the role of Toll, IMD and JAK-STAT signalling in modulating the number and time of parasite clearance. Linked to IMD-Relish, the timing of ISC proliferation had a pivotal role in the ability of the fly to clear the parasite quickly and to keep numbers down. Furthermore, our results provide a framework to establish the evolutionary conserved component of the response of dipteran insects against kinetoplastids.

## Results and discussion

### A natural parasite for *Drosophila melanogaster*

We have isolated trypanosomatid parasites from *D*. *melanogaster* caught in the wild on fruit baits in and around Oxford, UK (see materials and methods). Following sequencing of 18S rRNA we concluded that these parasites belonged all to the same species namely, *Herpetomonas megaseliae* (99% sequence identity with strain accession number U01014) [26]. However, through the molecular redefinition of phylogenetic relationships in the *Herpetomonas* genus, *Herpetomonas megaseliae* is now included in *Herpetomonas muscarum* and henceforth we will call the isolated parasite *H*. *muscarum* [27]. In our hands, the prevalence of the infection in the wild was 5.01% in males (or 16 out of 212 male flies caught and identified as *D*. *melanogaster*), in accordance with reports of more extensive samplings [28]. The prevalence in females was approx. 6% (or 5 out of 91). Following species identification, males were assayed directly for the presence of the parasite and were used to isolate it. Female flies were kept to establish separate lines and verify inect species identification (see materials and methods).

**Fig 1:**
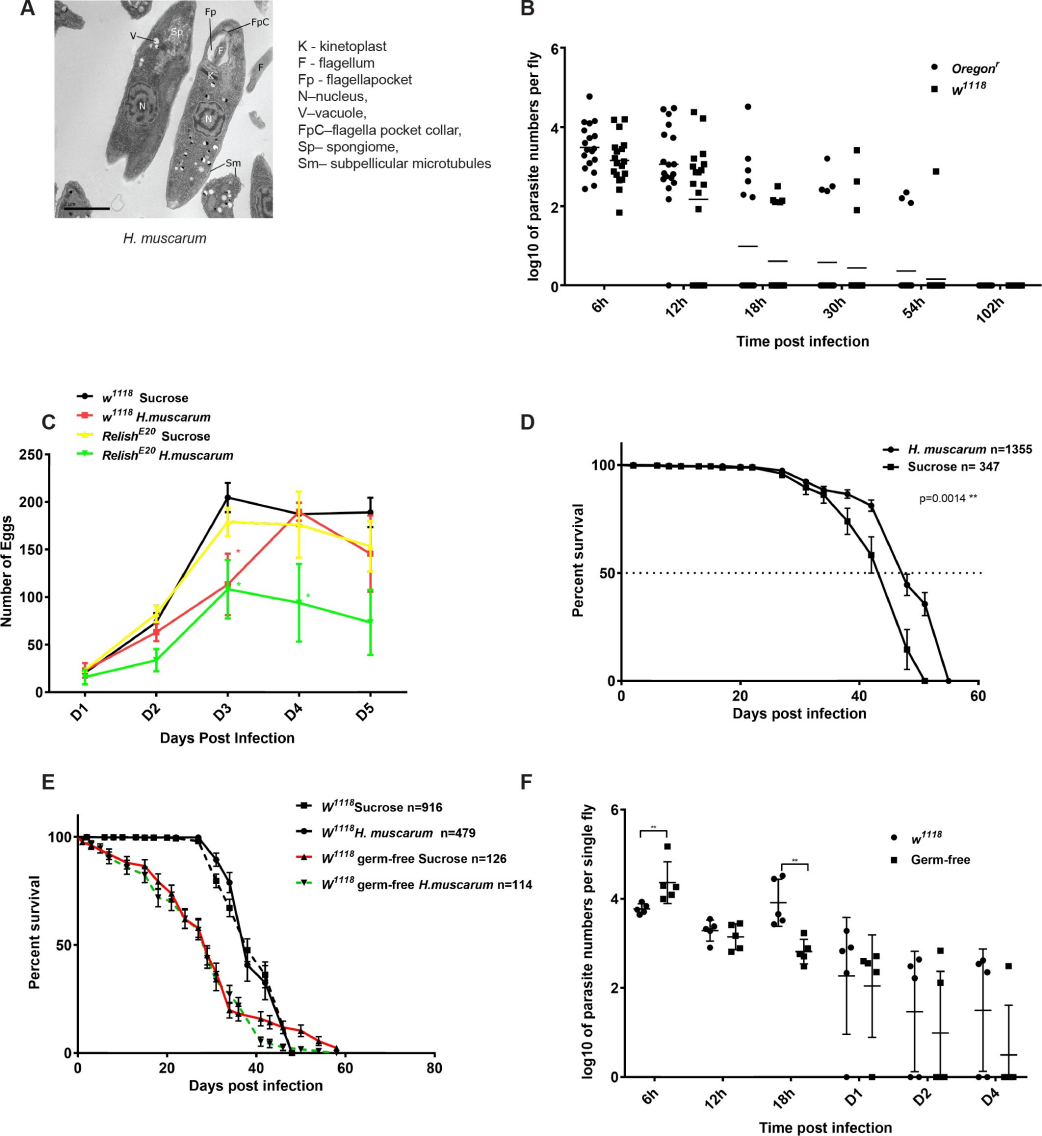
Infection of *Drosophila melanogaster* with its natural parasite *H*.*muscarum*. **(A)** EM of *H*. *muscarum* from culture. **(B)** Both *Oregon*^*R*^ and *w*^*1118*^ flies took up to 3 days to clear the parasites after an initial 6h oral feeding infection. **(C)** Fecundity assays of *w*^*1118*^ and *w*^*1118*^*; relish* flies where infection reduced egg laying in both strains compared to sucrose-only treatment **(D)** Median and maximum life span of *Oregon*^*R*^ flies was modestly (but significantly) increased after *H*. *muscarum* parasite oral infection compared to sucrose control. **(E)** Life span of *w*^*1118*^ flies infected with *H*. *muscarum* was statistically indistinguishable compared to sucrose-only fed controls either conventionally reared or germ free. **(F)** In the absence of gut microbiota, more parasite intake was observed during the first 6 hours of infection compared to conventionally reared flies. However, at 18h post-infection germ-free flies exhibited significantly reduced parasite numbers. Two-way ANOVA was used to analyse all data. For fecundity assays, statistical difference was observed on D3 between *w*^*1118*^ sucrose fed controls and *w*^*1118*^ infected flies and on D3 and D4 between *Rel*^*E20*^ sucrose fed controls and *Rel*^*E20*^ infected flies. On D4, *w*^*1118*^ sucrose fed controls, *w*^*1118*^ infected and *Rel*^*E20*^ sucrose fed were statistically indistinguishable and significantly different from *Rel*^*E2*0^ infected flies.

We were able to culture the parasite (see materials and methods; Fig 1A) and therefore wanted to transfer this host-parasite system in the lab to determine various aspects of this interaction. Pioneering work by Rowton and McGee has previously characterized infection of *Drosophila* laboratory populations by *Herpetomonas ampelophilae* [23, 24]. However, the experimental design did not preclude the infection of uninfected flies at different time points after the initial infection event. We therefore began by characterizing the likelihood, time course and fitness consequences of *H*. *muscarum* infection in laboratory *D*. *melanogaster* after a single exposure event. The infection protocol and scheme of time point sample collection for downstream experiments is illustrated in S1A Fig.

### Effects of *Herpetomonas* infection on *Drosophila* survival and life expectancy

We were able to record 100% infection rates in *D*. *melanogaster* laboratory flies (*Oregon*^*R*^ and *w*^*1118*^) fed with log-phase (72h) *H*. *muscarum* in 10% sucrose (S1B Fig). In this and all subsequent experiments, control flies are those fed with just 10% sucrose. Parasite presence was confirmed by visual inspection using live stains in the posterior (6h post-infection; S1C Fig) and anterior midgut (24h post-infection) (S1D Fig). These positions were reminiscent of the “swim back” of Leishmania towards the mouthparts in sand flies after digestion of the blood meal [8]. The possible attachment of *Herpetomonas* to the intestinal epithelium from the inside of the peritrophic matrix was also documented using infection with pre-stained parasites (S1C Fig). Finally, the dynamics of infection was followed using quantitative real time PCR in reference to a standard curve that was made each time from a fresh parasite culture used in that specific infection experiment (S1E Fig). Infection dynamics showed that *Oregon*^*R*^ and *w*^*1118*^ flies were able to clear infection within 4–5 days (Fig 1B).

Parasite infection reduced egg-laying of *w*^*1118*^ and *w*^*1118*^*; relish* mutant flies (Fig 1C) as well as *Oregon*^*R*^. This suggested a reduction in fecundity that was strategically placed to divert resources to parasite clearance [reviewed in 29]. Connected to the reduced energy spent for egg laying, *Oregon*^*R*^ flies showed a statistically significant increase in their median lifespan when infected with the parasite compared to controls (Fig 1D) while lifespan of infected *w*^*1118*^ flies was statistically indistinguishable from controls (Fig 1E). In germ-free conditions, *w*^*1118*^ flies exhibited a higher initial parasite uptake (6h post infection) but later inhibited parasite proliferation, especially at 18h post-infection (Fig 1F). Overall however, there was no difference in the lifespan of germ-free *w*^*1118*^ when fed sucrose only (control) or parasite in sucrose (Fig 1E).

Infected flies were not able to pass the parasite to non-infected adults when infected and non-infected flies were co-cultured in cages. This result showed that the parasite was able to survive inside one fruit fly host but could not subsequently transfer to an uninfected fly in contrast to *H*. *ampelophilae* [23]. In the latter case, transmission to adults and larvae was probably accomplished by feeding on substrates contaminated with host feces or “social digestion” of adult or larval cadavers by developing larvae. Our results however, leave open the possibility that flies killed all parasites prior to clearance or that an intermediate plant host is necessary for transmission, as some *Herpetomonas* species have been proposed to be associated with tomato plants [23]. Fast clearance and/or an intermediate plant host may also explain the low prevalence of infected fruit flies in the wild [30].

### Transcriptomic analysis of host response to parasite infection

**Fig 2:**
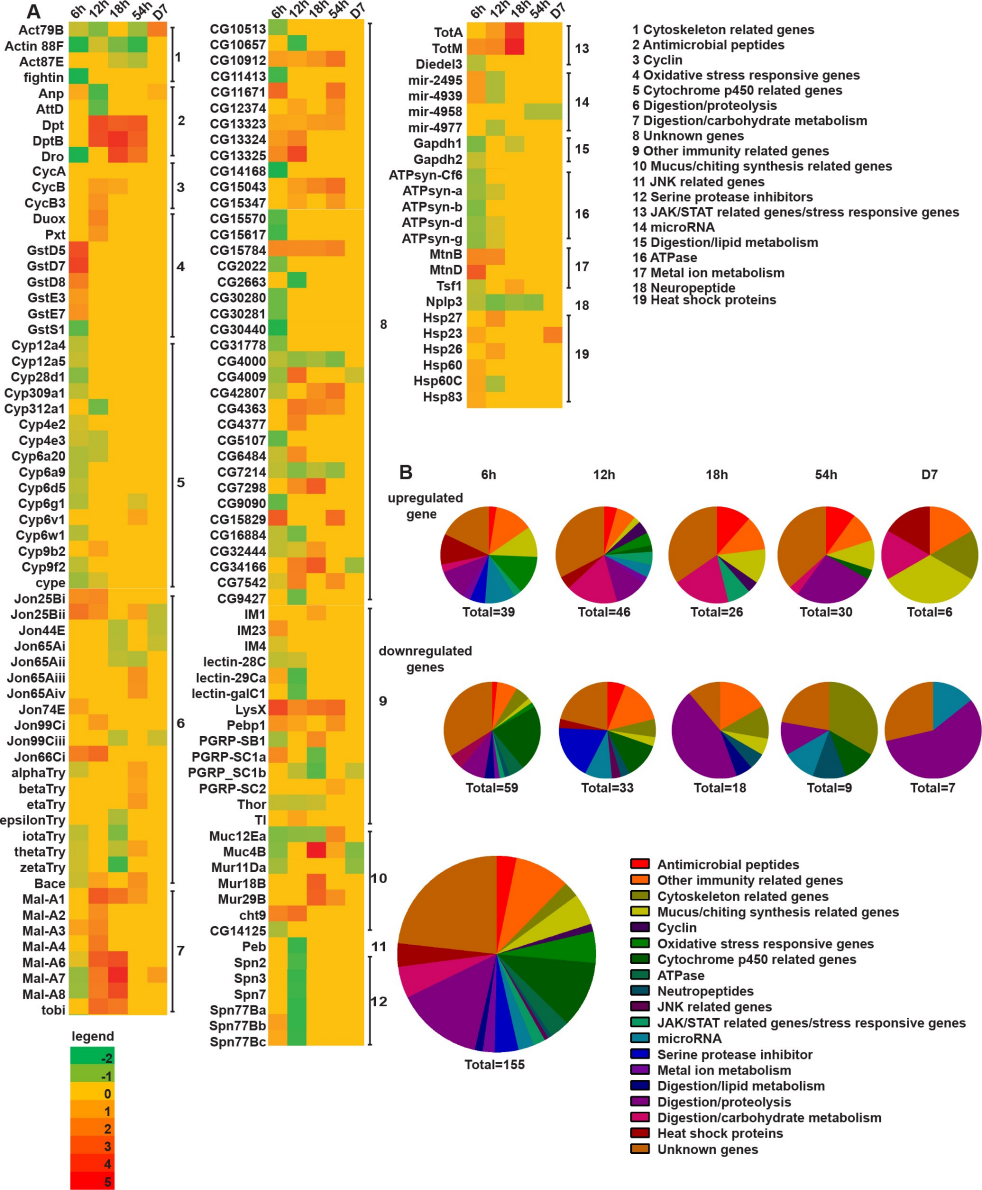
Host transcriptomics analysis of *H*. *muscarum* infection reveals the dynamics and gene networks involved. **(A)** Heat map showing the list of significantly regulated transcripts (>log2) compared to the corresponding sucrose control at each time point over the course of parasite infection. The list underlines the systemic temporal and spatial regulatory networks in response to *H*.*muscarum*. **(B)** This can be seen more clearly when the list in A is transformed into a pie chart comparing percentage, gene numbers and gene ontology of transcripts both upregulated and downregulated over the time course of infection.

We next sought to determine gene expression that was specifically altered by the presence of the parasite. We investigated transcriptome variations in whole, sucrose-only fed and sucrose-parasite fed flies following the same infection protocol shown in S1A Fig. Transcriptome data were generated using the Illumina RNA sequencing platform of sequencing poly-A RNA (thus avoiding bacterial contaminants) to capture both known and novel coding and noncoding RNA (see materials and methods). Taking into to account that the process of parasite clearance took about 105 hours (see Fig 1B) we followed infection dynamics of 4-day old flies from early time points (6, 12, 18h post infection), to 54h (as a mid-point) and finally to day 7, a time point after parasite clearance had been achieved. There were 1,556 genes that were significantly regulated following parasite infection (P<0.05). From these, our analysis identified 155 genes whose expression varied by at least a 2log fold change relative to expression in 10% sucrose-only fed flies (Fig 2A). The statistical confidence P-value was established in each of the three biological repeats. During the lifetime of infection those 155 genes included 66 upregulated-only transcripts, 56 downregulated-only transcripts and 33 (or 20%) that were upregulated in certain time points while downregulated in others. In terms of gene expression, the majority of the transcripts were differentiated at the initial stages of the response (98 and 79 at 6h and 12h following infection) while as the response progressed there was less differential transcription (39 transcripts at 54h and 13 at Day 7, see Fig 2B).

Using a global classification of gene ontology (GO), nearly a quarter (23.8%) of the genes were assigned as “unknown function” (11.6% upregulated-only, 8.38% downregulated-only and 3.82% both). Of those, the ones with at least one homologue could be divided into three main categories namely, intracellular signalling molecules, collagen-like cuticle proteins and genes with unknown function (see full data set and all gene expression tables at the European Nucleotide Archive website, project accession number PRJEB30020; https://www.ebi.ac.uk/ena/data/view/PRJEB30020). The rest of the transcripts were assigned to 17 functional categories (Fig 2B). These included digestion related to proteolysis and lipid metabolism, oxidative stress responsive and serine protease inhibitors (all upregulated mostly at 6h post-infection), metal-ion metabolism (upregulated at 54h but suppressed at 18h and Day 7 post-infection), antimicrobial peptides (AMPs) and other known immune-related genes (including known pathways and stress responsive genes; gradually upregulated through to the mid-point at 54h post-infection), mucus/chitin synthesis related and cytoskeletal genes (upregulated at 54h and Day 7 but suppressed at earlier time points), neuropeptides (suppressed at all time points) and micro-RNAs (activated at 6h, 12h and 18h but suppressed at 54h and Day 7). Based on this analysis we concluded that parasite infection triggers high levels of transcriptional signatures of signalling associated to immune, stress and metabolic responses. In this work, we study the involvement of immune response genes.

### Immune pathways related to *Herpetomonas* infection in *Drosophila*

We found that following parasite infection, there were several differentially expressed AMP genes that were targets of the IMD pathway, genes that coded for components of the oxidative stress response as well as the Toll receptor itself. Therefore, we addressed the functional relevance of these findings using the GAL4-GAL80^ts^–UAS-RNAi system where we could knock-down genes in adulthood in a ubiquitous or tissue specific manner avoiding any developmental or long-term effects. At the permissive temperature (18°C) GAL80^ts^ prevents GAL4 binding and therefore RNA-interference, whereas shifting to the restrictive temperature (30°C) inactivates GAL80, releases GAL4 and thus induces RNAi [31]. We tested that the system was effectively induced using three GAL4 lines (see below): one expressed in all immunocompetent tissues (*J6-GAL4* or *c564-GAL4*; S2A Fig), one expressed in enterocytes (*NP1-GAL4*; S2B Fig) and one expressed in ISCs and EBs (*esg-GAL4;* S2C Fig). The latter we also tested just at 18°C with infection vs. control to make sure that any difference we saw at 30°C was due to triggering RNAi and not a background effect. Indeed there was no expression of RFP in either infected or control guts at 18°C (S2D Fig).

### Antimicrobials

**Fig 3:**
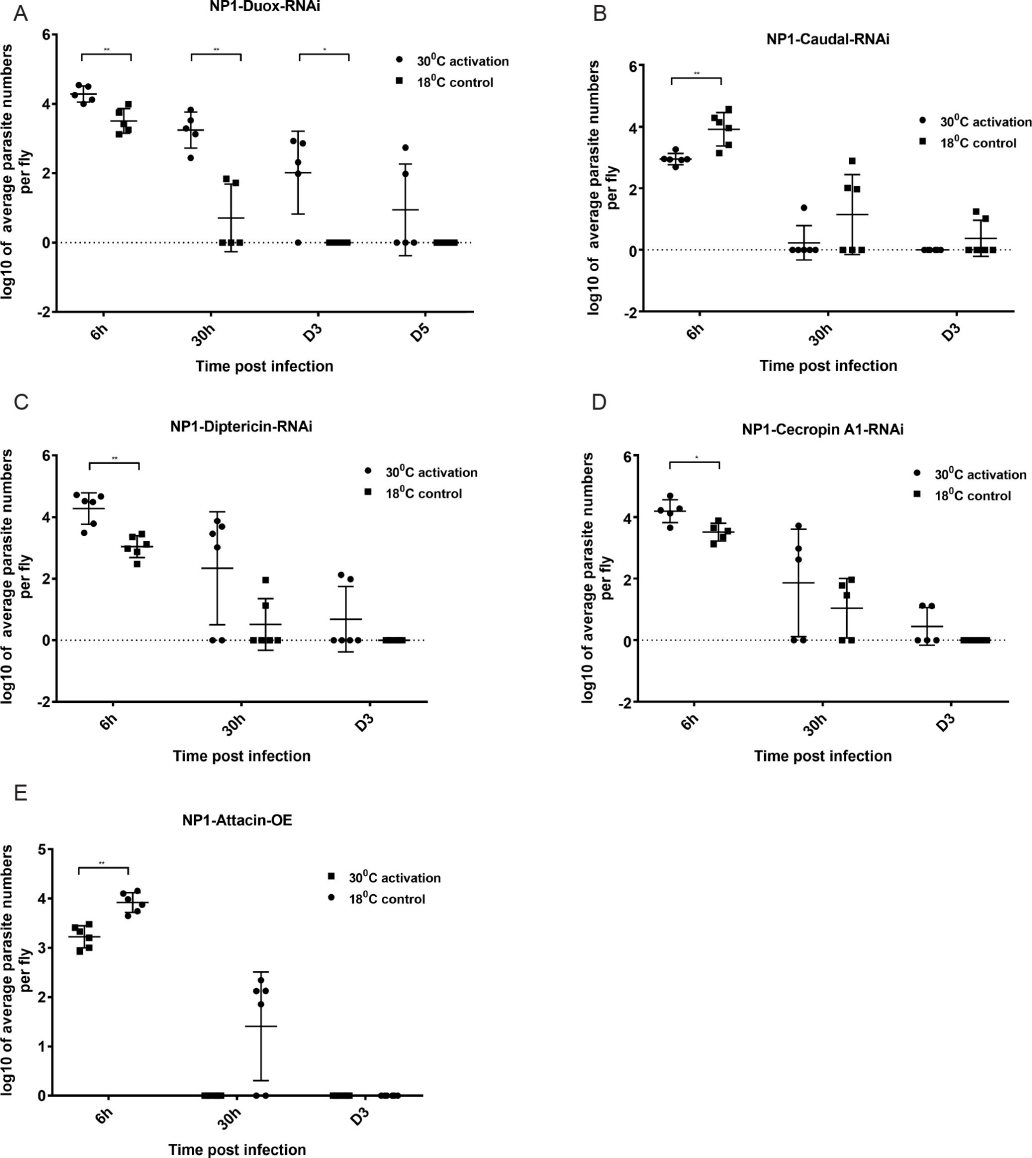
Influence of Reactive Oxygen Species (ROS) and AMPs on *H*. *muscarum* intake, infection dynamics and clearance. **(A)** Activation of the GAL4/GAL80^ts^ system and silencing of Duox (needed for ROS production) increased parasite numbers and delayed clearance beyond D5. **(B)** De-repressing transcription of a number of AMPs by knocking down *caudal* in ECs, reduced the parasite number intake and shortened the time for the parasites to be cleared. **(C)** Knocking down in ECs of *diptericin* (Dpt), a target of IMD pathway, increased the parasite number intake and but not the clearance time. **(D)**. RNAi of the AMP gene *Cecropin* in ECs (NP1-GAL4) also increased the initial parasite load. **(E).** In contrast, overexpression of the AMP gene *Attacin* in ECs cleared parasite infection in less than 30h. Two-way ANOVA was used to analyse all data (*p = 0.1, **p = 0.001, ***p = 0.0001).

We found that silencing the gene coding for Dual Oxidase, the enzyme responsible for ROS synthesis in ECs [32], significantly increased initial loads (6h post infection) and delayed parasite clearance (Fig 3A). Similarly, silencing *caudal* loss of which de-represses all Relish-regulated AMPs in ECs [33], significantly decreased parasite uptake (Fig 3B). Conversely, silencing the AMPs *Diptericin* (Fig 3C) or *Cecropin* (Fig 3D) increased uptake and delayed clearance while overexpressing *Attacin* had the opposite effect (Fig 3E). These results are consistent with a role for ROS as well as AMPs (especially those transcriptionally regulated by Imd/Relish) in initial uptake and clearing infection.

### Relish

**Fig 4:**
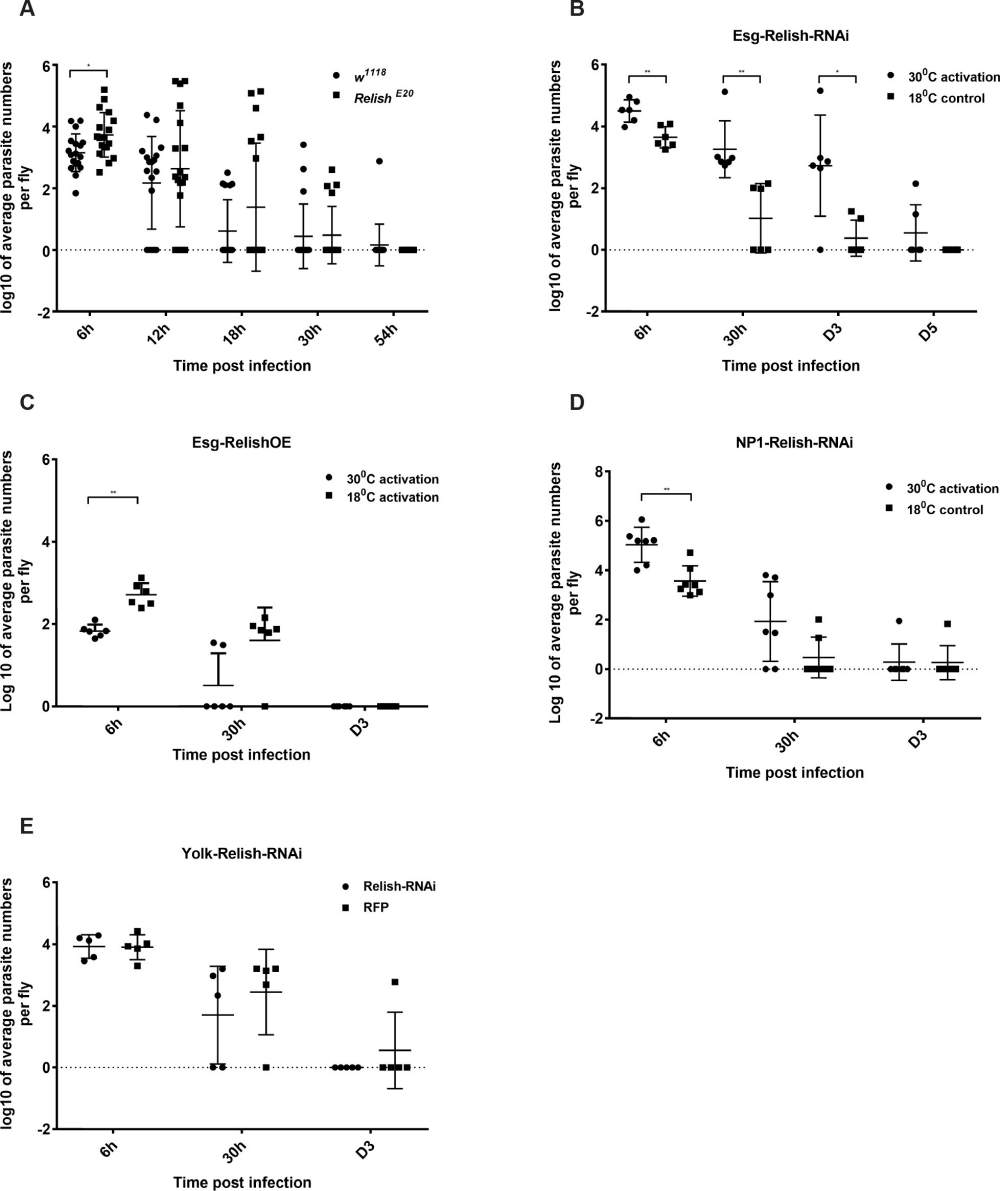
The influence of Relish on *H*. *muscarum* intake, infection dynamics and clearance in immunocompetent tissues. **(A)** Flies with loss of function of Relish (*Relish*^*E20*^), showed significant increase in parasite number intake only at 6h following infection. **(B).** A significant increase in both parasite numbers and clearance time was observed when Relish was blocked in ISCs. **(C)** Conversely, overexpression of Relish in ISCs significantly decreased parasite intake. **(D)** In contrast, a modest increase in parasite number intake was observed when Relish was blocked in ECs while **(E)** no effect when Relish was blocked in the fat body was observed. Two-way ANOVA was used to analyse all data (*p<0.01, **p<0.001, ***p<0.0001).

Consistent with the results above, *Rel*^*E20*^ flies displayed significantly higher parasite numbers at early time points (6h, 12h) compared to controls (Fig 4A). In exploring the tissue-dependency for Relish in the control of parasite numbers, we found that silencing Relish in progenitor cells (ISCs and EBs) with *esg-GAL4* had a significant influence in parasite numbers throughout the lifetime of the infection and delayed parasite clearance by 48h (Fig 4B). Conversely, overexpression in ISCs and EBs of a form of Relish that constitutively localises inside the nucleus [35], significantly decreased initial parasite loads and accelerated clearance with most flies clearing *Herpetomonas* at day 1 (Fig 4C). Therefore, Relish was necessary and sufficient to control parasite numbers in progenitor cells. Finally, silencing Relish in ECs significantly increased initial uptake only (Fig 4D) whereas no effect was observed when *rel* was silenced in the fat body (Fig 4E).

### Toll

**Fig 5:**
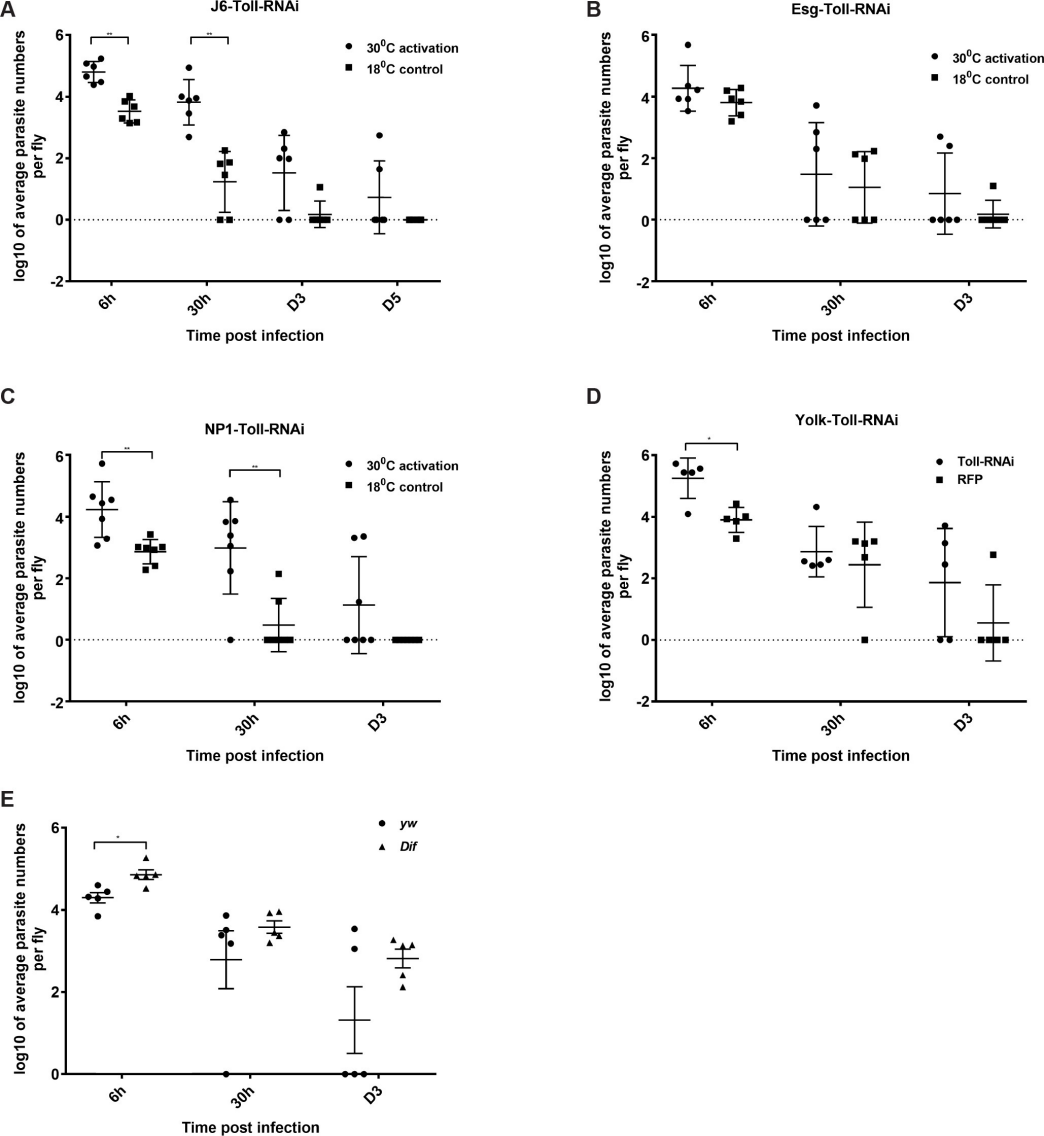
The influence of Toll on *H*. *muscarum* intake, infection dynamics and clearance in immunocompetent tissues. **(A)** Ubiquitous knocking down of Toll significantly increased parasite intake and slowed down clearance. **(B)** Knocking down of Toll in ISCs did not significantly influence parasite intake or clearance time. **(C)** In contrast, knocking down of Toll in ECs increased the parasite intake and clearance time. **(D)** Absence of Toll in the fat body showed significant increase in parasite intake compared to the RFP control. **(E)** Downstream of Toll, *dif* flies exhibited significantly increased parasite intake as well as delayed clearance. At the end of the observation period (D3) none of the *dif* flies had cleared the parasite. Two-way ANOVA was used to analyse all data (*p<0.01, **p<0.001, ***p<0.0001).

Next, we wanted to identify the role of the Toll receptor, which was differentially expressed in parasite-infected flies. Silencing Toll in all immunocompetent tissues significantly increased initial and early parasite load (6h to 1 day post-infection) (Fig 5A). In contrast to Relish however, we found that Toll was not required in progenitor cells (Fig 5B) but in ECs (Fig 5C). In addition, silencing Toll expression in the fat body (using *yolk-*GAL4) indicated that Toll activity was also important there but only for controlling initial parasite uptake (Fig 5D). To corroborate the role of the Toll pathway in controlling parasite numbers, we tested *dif*^*1*^, a mutant in DIF [11]. We found that *dif*^*1*^ mutant flies displayed a significant increase in the initial *Herpetomonas* uptake (Fig 5E). Moreover, *dif*^*1*^ flies were not able to clear the infection at D3 in comparison to their *yw* genetic background (Fig 5E).

### STAT

**Fig 6:**
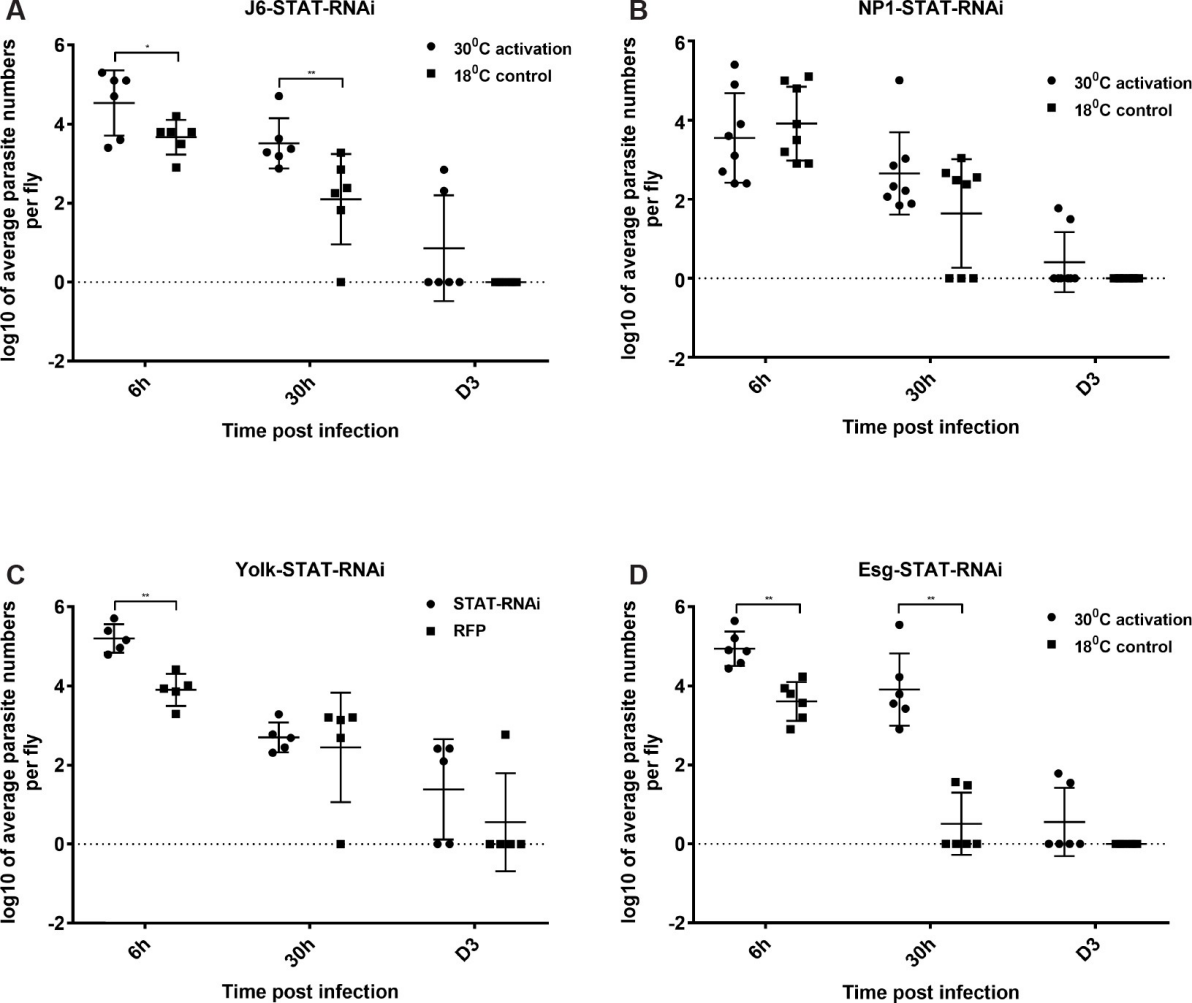
JAK-STAT signaling in *H*.*muscarum* intake and clearance. **(A)** Knocking down of STAT transcription factor in all immunocompetent tissues significantly increased parasite intake and slowed down clearance. **(B)** In contrast, knocking down STAT in ECs did not influence parasite intake number or clearance time. **(C)** This was also the case when STAT was knock down in the fat body where only parasite numbers at the earliest time point of 6h were increased. **(D)** The most significant effect was observed when STAT was silenced in the ISCs, significantly increasing both parasite number intake and clearance time. Two-way ANOVA was used to analyse all data (*p<0.01, **p<0.001, ***p<0.0001).

Targets and signaling components of the JAK-STAT were also differentially regulated following parasite infection. As the pathway has been implicated in gut physiology and immunity [16], we investigated the effect of silencing STAT in ECs, progenitor cells, hemocytes and fat body. Using J6-GAL4 we found that silencing expression of the Stat transcription factor (Stat92E) increased initial parasite numbers (6h post infection) and delayed clearance (Fig 6A). However, there was no requirement for STAT in ECs (Fig 6B) or the fat body (Fig 6C). However, silencing Stat in progenitor cells resulted in a significant difference in both parasite numbers in the early phase of infection (6h to day 1) and delay in clearance at day 3 (Fig 6D). This result, along with the fact that Stat has been shown to be expressed in ISCs (see below) indicated that Stat activity was required in progenitor cells to control parasite numbers and clearance.

### Parasite infection increases Stat-mediated transcription and proliferation of ISCs

**Fig 7:**
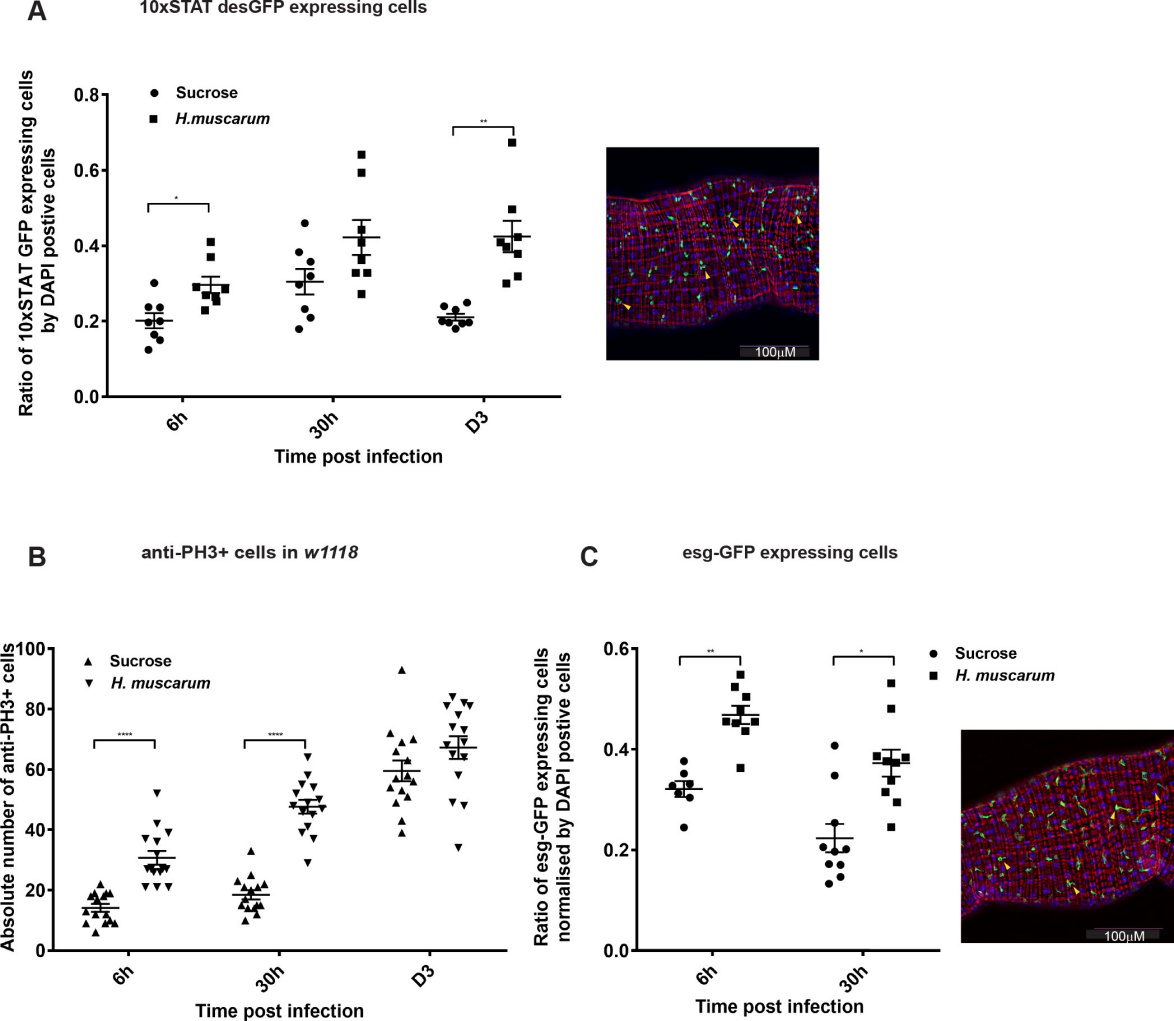
STAT activation & ISC proliferation following *H*.*muscarum* infection. **(A)** Increased JAK-STAT activity could be observed and quantified by measuring the GFP-expressing cells in the gut following parasite infection with a reporter line where GFP was under the control of 10 copies of STAT binding sites (*10xSTAT-desGFP*). A selected region of the midgut at 20x magnification is shown (right panel). Yellow arrows indicate the GFP cells co-localizing with DAPI which were counted. Quantification of GFP expressing cells normalized with DAPI from 12 guts is shown (left panel). Results in the graph are from three independent infections. Following parasite infection, the number of GFP-expressing cells showed significant upregulation at 6h and D1 post infection. **(B)** The number of proliferating ISCs stained by anti-histone 3 (anti-PH3) antibody in infected *w*^*1118*^ flies, showed significant increase at 6h and 30h post infection. **(C)** Increased number of ISCs and EBs following parasite infection at day 1, could be observed and quantified by measuring GFP expressing cells in the gut of *esg*-CD8-GFP, *UAS-Gal4*, *tub-Gal80*^*ts*^ flies after an initial 6-day incubation at 30°C (infection and subsequent culture at 25°C). A selected region of the midgut at 20x magnification is shown (right panel). Yellow arrows indicate counted GFP cells co-localizing with DAPI. Quantification of GFP expressing cells normalized by DAPI from between 7 and 11 guts for each time point and three independent infections is shown in the graph (left panel). Two-way ANOVA was used to analyse all data (*p<0.01, **p<0.001, ***p<0.0001).

The involvement of JAK-STAT signalling in ISC proliferation and intestinal regeneration upon Upd cytokine secretion from ECs, has been documented previously [19, 34]. Using multimerized Stat92E consensus binding sites, which control expression of destabilised-GFP (10x-STAT-GFP) as a reporter of JAK-STAT signalling activity [36], our results indicated the involvement of STAT in parasite infection. Following parasite feeding, GFP-expressing cells were transiently increased early, at 6h and day 1 post infection compared to the sucrose-only control but there was no difference at day 3 (Fig 7A). This correlated with a significant increase in proliferative cells measured with an antibody against phospho-histone-3 (PH3) in *w*^*1118*^ flies (Fig 7B quantification). Moreover, there was persistent increase in GFP-positive progenitor cells (6-30h) when an *esg-GAL4*, *UAS-GFP* strain was infected with *Herpetomonas* (Fig 7C).

### ISC proliferation and *Herpetomonas* infection

**Fig 8:**
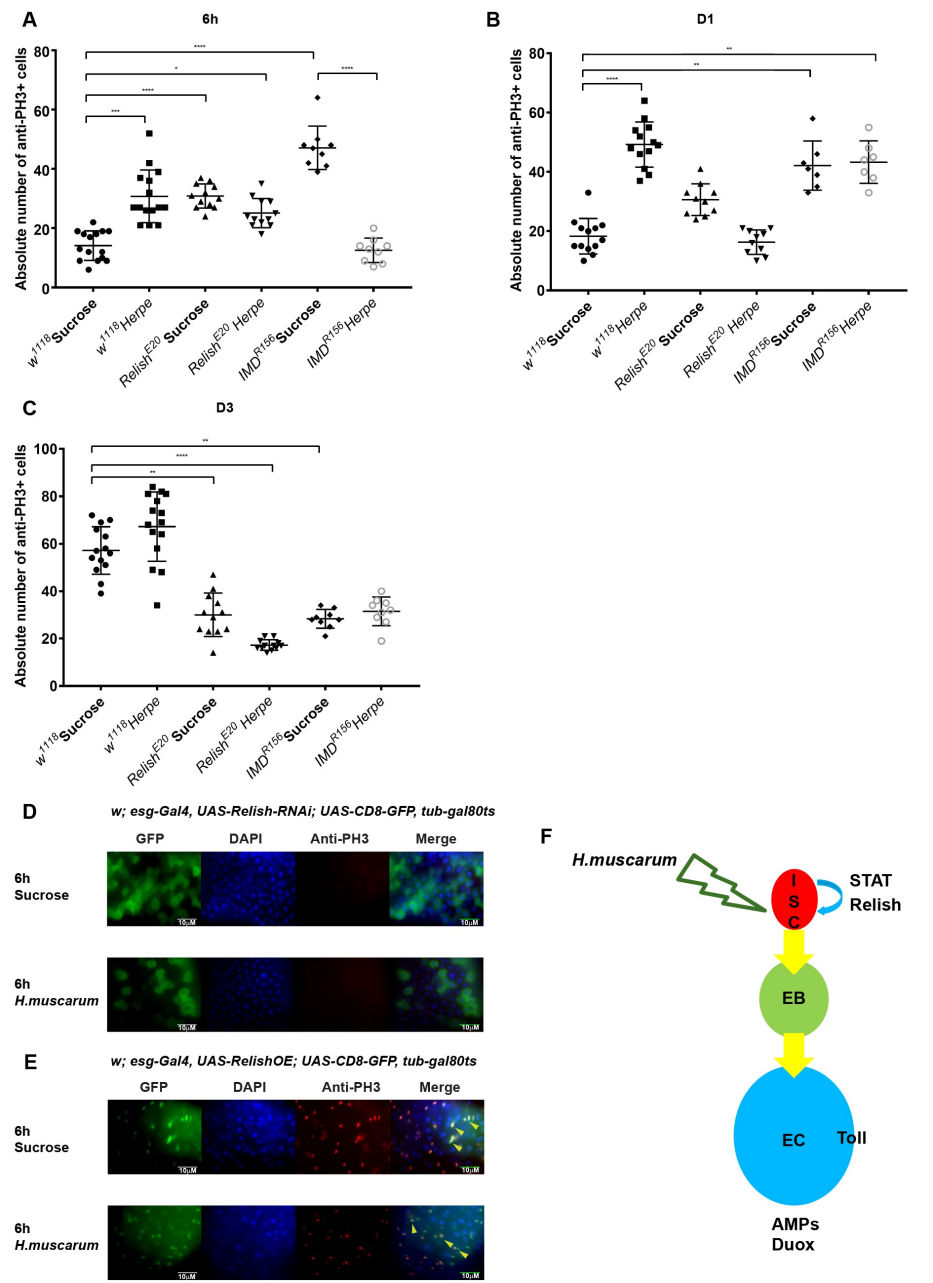
ISC proliferation and parasite infection. ISC proliferation dynamics following *H*.*muscarum* infection at 6h **(A)**, Day 1 **(B)** and Day 2 **(C)** post-challenge; the parasite was able to suppress ISC proliferation in the absence of Relish (6h, D1 and D3) and to a lesser extend Imd (6h). **(D)** Relish also played a role in the proliferation of ISCs as loss of Relish blocked ISC proliferation (PH3-red channel) following parasite challenge while **(E)** Relish overexpression induced ISC proliferation even in the absence of the parasite (see PH3 in sucrose control) **(F)** A cartoon illustrating a working model of the *H*. *muscarum* oral infection model in *Drosophila*. Both STAT and Relish transcription factors are essential for ISC proliferation and differentiation whereas Toll signaling as well as ROS generation (through Duox) is important in ECs. Parasite infection can also induce Relish-dependent transcription as seen in the upregulation of IMD controlled AMPs. Our hypothesis is that the combinatorial effect from STAT-mediated signaling and Relish-dependent transcription encourages a hyper proliferation of ISCs which eventually results in a fast epithelium turn over in the *Drosophila* gut. Such a mechanism, together with the expression of effector molecules like AMPs and ROS would be able to remove the parasite quickly and efficiently. Two-way ANOVA was used to analyse all data (*p<0.01, **p<0.001, ***p<0.0001).

Given the above, we next explored the signaling underlying proliferation of ISCs following parasite infection. When *w*^*1118*^ were infected with *H*. *muscarum*, PH3^+^ cells were significantly increased continuously compared to the sucrose control, starting from 6h (Fig 8A), day 2 (Fig 8B) and day 3 (Fig 8C). In contrast, sucrose-only fed controls showed a stable number of PH3^+^cells in both 6h (Fig 8A) and day 2 (Fig 8B) only increasing at day 3 (Fig 8C). The number of PH3^+^ cells in sucrose-only fed *imd*^*R156*^ was significantly higher than *w*^*1118*^ controls indicating a de-repression of progenitor cell proliferation in the absence of Imd (Fig 8A). Parasite infection significantly suppressed this phenomenon at 6h post-infection indicating a strategy from the parasite’s side to achieve midgut establishment (Fig 8A). Nevertheless, this suppression was not evident at day 2 (Fig 8B) or day 3 (Fig 8C). At these time-points, the numbers of PH3^+^ cells between infected and sucrose-fed *imd*^*R156*^ were statistically indistinguishable. Intestinal proliferation in *rel*^*E20*^ mutant flies (sucrose control) was also significantly increased compared to *w*^*1118*^ controls but not as pronounced as *imd*^*R156*^ (Fig 8A). As with *imd*^*R156*^, parasite infection of *rel*^*E20*^ significantly suppressed de-repression of intestinal proliferation (Fig 8A). The number of PH3^+^ cells in *rel*^*E20*^ infected intestines however, was kept significantly lower both at day 2 (Fig 8B) and day 3 (Fig 8C) compared to both infected *imd*^*R156*^ and controls. This indicated an important and more general role for Relish in ISC proliferation compared to Imd. When silencing *rel* in progenitor cells, the majority of the GFP expressing cells in the guts of *esg*-CD8-GAL4, UAS-GFP; GAL80^ts^ flies were enlarged GFP-positive cells that have lost their proliferative capacity as no PH3^+^ was detected (Fig 8D). Conversely, overexpression of Relish increased the population of smaller GFP expressing ISC-looking cells, which correlated with an increase in proliferating PH3^+^ cells (Fig 8E). This PH3^+^ proliferation was suppressed by the parasite (Fig 8E).

Thus, independently of infection, Relish was sufficient for intestinal progenitor cell proliferation. High levels of Relish in the nucleus pushed cells to be PH3^+^, with an ISC morphology whereas low levels of Relish provoked progression in the progenitor lineage to larger EBs. The role of Relish in proliferation of midgut progenitor cells and the fact that its loss resulted in sustained low numbers of PH3^+^ cells following infection, suggested an explanation for the delay in parasite clearance in *rel*^*E20*^. In addition, STAT-mediated transcriptional activity correlated with ISC proliferation following infection and was important for parasite clearance. Finally, AMPs, Duox/ROS and Toll were required in ECs (Fig 8F).

Nevertheless, even in the absence of Relish the parasite was eventually cleared. This suggested that gut epithelium renewal was just one of the factors influencing parasite clearance and that most likely “flushing” of the parasite through the mechanical contraction of the gut was at play. Clearance by “flushing” would be possible if the parasite was failing to attach to the gut epithelium. More work is needed to define a possible interaction interface for this attachment.

*Herpetomonas* infection induced an acute early increase in ISC proliferation in *w*^*1118*^ control flies as seen with PH3^+^ quantification (Fig 8A). However, this was suppressed in immune-deficient *rel*^*E20*^ or *imd*^*R156*^ flies even though these flies displayed a higher than the control ISC proliferation in sucrose-only treatment (Fig 8A). This suggested that the parasite actively suppressed proliferation of ISCs and epithelial renewal as a means of establishing a stable midgut presence and the Imd/Relish pathway was paramount to resist this strategy. This has been also observed in *Vibrio cholera* [37] and *Pseudomonas entomophila* [38] intestinal infections where delamination of ECs is an important way with which “shedding” of cells removes the pathogen as well. It would be interesting to see whether suppression of ISC proliferation happens in the intestines of tsetse infected with trypanosomes or sand flies infected with *Leishmania*. Intriguingly, PH3^+^ suppression following infection was most pronounced in *rel*^*E20*^ where the number stayed significantly lower than the control for the duration of our experiment (day 3, Fig 8C). This was in contrast to *imd*^*R156*^ where numbers were significantly low early on (6h) but were indistinguishable to controls from day 1 onwards (Fig 8B and 8C). This suggested a more important role for Relish than Imd in ISC proliferation and does not conform to a linear pathway hypothesis. Therefore, Relish may regulate ISC proliferation by integrating signals from other signalling networks in addition to Imd.

### Conclusions

We established for the first time, a framework to systematically study how the model dipteran insect *Drosophila*, responds to a trypanosomatid parasite infection. We showed that Relish, STAT, Toll, Duox and AMPs were all important for fast clearance and control of parasite numbers. Our results provide cellular context to similar data from tsetse where RNAi of Relish or of an AMP homologous to *attacin* increased *T*. *brucei* numbers in infected flies [39]. However, more work is needed to ascertain whether the *Drosophila-Herpetomonas* system could be used to address questions in insect vectors (e.g. tsetse, sand flies) where the tool-box available for direct functional studies is not yet fully developed.

## Materials and methods

### *Drosophila* collection and species identification

We collected *Drosophila* in a residential area using the corresponding author’s back garden and compost tip (inside the garden) as places to trap fruit flies. As such, no consent was necessary. Collection was done through 5-day periods from late March to late June 2011. During this period, traps containing fermented banana were set every week. The nine species of the *melanogaster* species subgroup are morphologically very similar but male genitalia is a reliable characteristic to distinguish males [28]. For females we used as a discriminatory characteristic the area below the eye (cheek) along the long axis, which is broader in *D*. *melanogaster* than all other members of the group [40]. To confirm our identification following establishment of separate stocks from a single female founder we randomly selected flies identified morphologically and used a PCR diagnostic test for the antimicrobial peptide gene drosomycin as described [22]. Species identification was confirmed with first generation offspring.

### *Drosophila* stock

Single trapped females were isolated in vials and their offspring cultured as separate “isofemale” lines to verify species identification [28]. Trapped males were used to isolate and culture the parasite. Starting from a single cross (one female-one male), *Oregon*^*R*^ flies were used as a wild type laboratory strain, for establishment of laboratory oral feeding infection protocols and transcriptomics experiments thus ensuring a less variable, streamlined genetic background. *w*^*1118*^ (BL #6326) and *yw*^*67c23*^ (BL #6599), were used as controls and for the genetic background of all the other strains used in these studies and were obtained from the Bloomington Stock Centre. IMD, Toll and STAT signaling pathway mutants: *yw*^*67c23*^,*Dredd*^*B118*^ (BL #55712); *w*^*1118*^*;relish*^*E20*^ (BL #55714) and *y*^*1*^ w*; Dif^*1*^ *cn*^*1*^ *bw*^*1*^ (BL #36559) were also obtained from the Bloomington Stock Centre. The GAL4 driver lines used were: *w*^*1118*^; *np1-GAL4* (ECs) was kindly provided by Heinrich Jasper (Buck Institute for Research on Aging, Novato, CA, USA), *yolk-GAL4* (female fat body), *w*^*1118*^*; P{GawB}c564* (*J6-GAL4*, expressed in fat body, gut, hemocytes, (BL#6982) and *esg-GAL4* (expressed in ISCs and EBs) was obtained by Bruno Lemaitre EPFL, Lausanne see ref [19].

All the *w*^*1118*^, *UAS-RNAi* transgenic lines were purchased from Vienna *Drosophila* Resource Centre (VDRC). *GAL80*^*ts*^ strains were all obtained from Bloomington Stock Centre: P*{tubP-GAL80ts}Sxl*^*9*^, w*/FM7c (BL#7016), w*; P{tubP-GAL80ts}^*2*^*/TM2 (BL#7017)*, w*; sna^*Sco*^*/CyO; P{tubP-GAL80ts}*^*7*^ (BL#7018) and w*; P{tubP-GAL80ts}^20^; TM2/TM6B, Tb^1^ (BL#7019). Appropriate lines were used to cross with the *GAL4* driver lines to ensure that the *GAL4* and *GAL80*^*ts*^ genes were bred onto different chromosomes into homozygosity before being used in RNAi screening and other breeding schemes. We also used *10xSTAT-desGFP* [36].

### Culture of *H*.*muscarum* and EM

The isolated *H*.*muscarum* used in the study was routinely cultured in 10% FBS (Gibco) supplemented *Drosophila* Schneider’s-2 (S2) media (Sigma-Aldrich). The parasite culture was maintained by sub-culturing every 3 days at 1:100 dilution to a fresh 5 ml S2 media. To count the parasite cells, cultures were first spun down at 2,000rpm for 5 mins and the cell pellets were treated with 10% methanol for 15 minutes to kill and immobilize the parasite cells. The cells were subsequently precipitated by spinning at 2,000rpm for 5 mins and washed twice by 1xPBS. After washing, cell pellets were resuspended in 1XPBS and diluted by 100 times in PBS before being transferred to a hemocytometer to count under a bright field light microscope at 100x magnification.

### TEM Microscopy of *H*. *muscarum* cells

5ml of culture (10^6^ cells/ml) was fixed using 2ml of 16% PFA (w/v) and 0.8ml of 25% glutaraldehyde (v/v) at 28°C. Cells were pelleted at 10000rpm for 5min and the fixative removed. Cell pellets were washed twice in 1ml 0.1M PIPES pH 7.2 and once in 1ml 0.1M PIPES pH 7.2 with 50mM Glycine. The supernatant was removed, and the pellets embedded in 0.5ml 2.5% agarose (w/v). Embedded pellets were fixed in 1% OsO_4_ for 1h at 4°C, washed three times in 2ml milli-Q water and then incubated overnight in 0.5% Uranyl acetate in milli-Q water at 4°C. Following this, cell pellets were washed for 10 mins in 2ml of milli-Q water at room temperature on a rotor before a 30min incubation in 30% EtOH on ice. The pellets were then dehydrated by immersion in increasing concentrations of 2ml EtOH (50%, 70%, 80%, 90% and 95%) for 10 mins each on ice. The three final dehydrations were done for 30min in 2ml 100% EtOH. Pellets were then incubated in 2ml of the following mixes of (v/w) ethanol: agar100 resin; 3:1 (1h with rotation), 1:1 (2.5h with rotation), 1:3 (1h with rotation). Pellets were then incubated in 2ml 100% agar100 resin for 24 hours with the resin replaced with fresh resin at 18 hours and 22 hours. Finally, pellets were transferred into the base of beem capsules (Agar Scientific) and the resin set at 60°C for 18 hours. Images were taken using the FEI Technai 12 Transmission electron microscope with the Gatan Ultrascan 1000 CCD camera and Gatan Digital micrograph and SerialEM image acquisition platforms.

### Oral feeding infection and quantification of parasite by real-time PCR

For each independent infection of a group of 20–30 flies, 10e7 *H*. *muscarum* parasite cells were harvested from a 3 days-old culture (which showed the highest infectivity rate from our experience) and resuspended in 500ul 1% sucrose. The parasite solution was then transferred to a 21mm Whatman Grade GF/C glass microfibre filter circle (Fisher Scientific). Circles containing the parasite cells were placed into standard *Drosophila* small culture vial without any food. The flies used in the infections were 4–5 days old before they were starved overnight. After starvation, the flies were transferred to food vials that contained the Whatman circles with the parasite cells. After 6h of feeding, flies were moved and reared on standard yeast/molasses medium. At different time points post oral infection (including the 6h feeding as 6h point), infected flies were collected for downstream experiments.

An absolute quantification of real-time PCR based method was developed to quantify the parasite numbers in the flies. This was done every time an experimental infection was carried out as follows. *H*.*muscarum* parasite cells from 1ml of the 3 days old culture used for the infection, were harvested and genomic DNA (gDNA) was isolated. The purified gDNA was serially diluted and was used as template in RT-PCR reactions to obtain linear regression of gDNA verse Ct values using *H*. *muscarum* gene specific primers. The serial dilution of parasite gDNA was then correlated with the serial dilution of the absolute number of parasite cells from the same culture. Hence a standard curve of the parasite numbers versus the Ct values was established as shown (S1E Fig). Isolation of gDNA from infected whole flies was also carried out and used for RT-PCR measurements. We did not use any uninfected fly DNA (only sucrose fed controls) for RT-PCR as the primers were parasite gene specific and would not pick up any PCR products from uninfected flies. For each individual fly or group of flies infected, gDNA was exacted (containing both fly and parasite gDNA). By measuring the Ct value of the gDNA sample, the absolute number of parasite cells could be extrapolated from the standard curve. The gDNA of the cells was extracted using cells and tissue genomic DNA isolation kit from Norgen Biotek no 53100 following the manufacturer’s instruction. *H*. *muscarum* gene specific primers used in the RT-PCR was designed based on the paraflagellar rod protein (PFR2) gene (GenBank accession no: AY785780). The primer sequences were: HmegRodF 5’-GGACTGCTGGAACAAGATC-3’, HmegRodR 5’-AGCTTCTTGTGCTGGGAG-3’.

### RNAseq and data analysis

Total RNA of 8–10 flies at 6h, 12h, 18h, 30h, 54h and D7 (S1A Fig) and post *H*. *muscarum* oral infection was extracted with total RNA purification kit from Norgen Biotek no. 17200 following the manufacturer’s instruction. Triplicate of samples were used for each time point. cDNA libraries were prepared with the Illumina TruSeq RNA Sample Prep Kit v2. All sequencing was performed on Illumina HiSeq 2000 plaftform using TruSeq v3 chemistry (Oxford Gene Technology, OGT). All sequence was paired-end and performed over 100 cycles. Read files (Fastq) were generated from the sequencing platform via the manufacturer's proprietary software. Reads were processed through the Tuxedo suite [41]. Reads were mapped to their location to the appropriate Illumina iGenomes build using Bowtie version 2.02. Splice junctions were identified using Tophat, version v2.0.9. Cufflinks was used to perform transcript assembly, abundance estimation and differential expression and regulation for the samples (version 2.1.1). RNA-Seq alignment metrics were generated using Picard.

At each corresponding time point, total RNA from triplicate of sucrose fed controls was extracted and the transcriptomes were sequenced and analyzed in the same manner for the subsequent comparison and data analysis. Differential expression of transcripts from three paired triplicates was statically analysed by Cufflinks. We have selected transcripts that showed log2 fold change below -2 or above +2 of absolute FPKM values (Fragments Per Kilobase of transcript per Million mapped reads) compared with sucrose fed samples to generate the heat map. An average of 26280113 paired-end reads were sequenced per sample. A total of 75.73 Gigabases (788403397 reads) of sequence data were read and aligned at high quality. The entirety of the RNA-seq data along with all the gene expression analysis tables can be found at The European Nucleotide Archive (ENA) with accession number PRJEB30020.

### Lifespan monitoring

Life span of flies infected or sucrose fed controls was monitored by counting the number of live flies on a 3-day interval until the last single fly in the culture died. During that time live flies were put in fresh food every 2 days.

### Generation of germ-free flies and RT-PCR to measure dynamics of gut microbiota

Germ free flies were generated following standard protocols [19]. Briefly, 200–300 embryos were collected from fly cages fitted with apple juice plates. The embryos were then treated with 50% bleach for 2–3 minutes until all embryos were dechorionated when observed under a stereoscope. Subsequently, embryos were washed twice with 70% ethanol followed with autoclaved miliQ water to remove residual ethanol. Treated embryos were then transferred to vials with standard yeast/molasses medium. Both vials and food were autoclaved forehand. From then on, aseptic procedures were employed in handling of vials with flies.

The transcriptional dynamics of gut microbiota was measured by qPCR using 16s rRNA primers. Total RNA from 8–10 flies was extracted using total RNA purification kit from Norgen Biotek no. 17200 following the manufacturer’s instruction. 16s rRNA primers were 27F 5’-AGAGTTTGATCCTGGCTCAG-3’ and 1492R 5’-GGTTACCTTGTTACGCTT-3’.

### Dissection of fly guts and immune-staining

Dissection of the fly guts and immune staining was generally following a standard protocol as in [41]. Rabbit anti Phospho-Histone H3 (Ser10) (anti-PH3) and goat anti rabbit Alexa Fluor A568 secondary antibody were both obtained from Invitrogen.

### Fluorescence microscope and image analysis

Images of the gut with GFP expressing cells, DAPI and anti-PH3 staining were obtained with either Zeiss Axioplan 2 (Carl Zeiss) or Zippy DeltaVision Elite (Applied Precision). Image J was used to quantify individual cells from the stacks taken by Zippy DeltaVision Elite. GFP expression cells and DAPI cells in the same designated region were counted separately and automatically. The relatively ratio of GFP expressing cells normalized by DAPI cell was used instead of absolute cell numbers to represent more accurately the change of the GFP expressing cells by eliminating factors such as physiological and anatomical change of the gut tissue in response to parasite infection and/or experimental handling.

## Supporting information

S1 Fig Laboratory protocols of *Drosophila* infection with *Herpetomonas muscarum*. **(A)** Infection protocol showing the time points for both RNA-seq as well as sampling for parasite numbers following various RNAi treatments. **(B)** Parasite infection was also verified by studying intestines manually at different time points. **(C)** Pre-stained parasites with DAPI (blue) and mitotracker (red) was seen in the anterior **(C)** and posterior midgut **(D),** where close-ups **(from C)** indicated the formation of rosettes reminiscent of Leishmania. **(E)** A representative standard curve that was made each time with the parasite culture used to infect, so as to help quantify absolute numbers of parasites in infection experiments.
(TIF)

S2 Fig Testing the conditions for RNAi with GAL80^ts^. **(A)** Using UAS-RFP to investigate the control of GAL80^ts^ over the GAL4 drivers used in this study namely, the general driver J6-GAL4 **(B)** the EC-specific driver NP1-GAL4 **and (C)** the ISC/EB-specific Esg-GAL4. **In all of these GAL4-GAL80**^**ts**^ **combinations RFP was only induced at 30**°**C. (D)** At the restrictive temperature (18°C) the system was not inducible following infection.
(TIF)

## References

[1] Hotez PJ. (2008) Neglected infections of poverty in the United States of America. PLoS Negl Trop Dis. 2, e256 10.1371/journal.pntd.0000256 18575621 PMC2430531

[2] Hotez PJ. 2009 The neglected tropical diseases and their devastating health and economic impact on the member nations of the Organisation of the Islamic Conference. PLoS Negl Trop Dis. 10 27;3(10):e539 10.1371/journal.pntd.0000539 19859530 PMC2760759

[3] Hotez PJ, Pecoul B. "Manifesto" for advancing the control and elimination of neglected tropical diseases. PLoS Negl Trop Dis. 2010 5 25;4(5):e718 10.1371/journal.pntd.0000718 20520793 PMC2876053

[4] Aksoy S, Lehane M and Levashina E (2004) Immune responses and parasite transmission in blood sucking insects Trends Parasitol 20: 433–439 10.1016/j.pt.2004.07.002 15324734

[5] Lehane MJ, Msangi AR (1991) Lectin and peritrophic membrane development in the gut of *Glossina m*. *morsitans* and a discussion of their role in protecting the fly against trypanosome infection Med Vet Entomol 5: 495–501. 1773127 10.1111/j.1365-2915.1991.tb00578.x

[6] Van Den Abbeele J, Rotureau B (2013). New insights in the interactions between African trypanosomes and tsetse flies. Front Cell Infect Microbiol 3, 63 10.3389/fcimb.2013.00063 24137569 PMC3797390

[7] Lehane MJ (1997) Peritrophic matrix structure and function Annu Rev Entomol 42: 525–50 10.1146/annurev.ento.42.1.525 15012322

[8] Bates PA (2008) *Leishmania* sand fly interaction: progress and challenges Curr Opin Microbiol 11: 340–4. 10.1016/j.mib.2008.06.003 18625337 PMC2675783

[9] Christensen BM, Forton KF, Lafond MM, Grieve RB (1987) Surface changes on Brugia pahangi microfilariae and their association with immune evasion in Aedes aegypti. J Invert Pathol 49: 14–18. 10.1016/0022-2011(87)90120-0 3794383

[10] Baton LA, Ranford-Cartwright LC (2005) How do malaria ookinetes cross the mosquito midgut wall? Trends Parasitol 21: 22–28. 10.1016/j.pt.2004.11.001 15639737

[11] Weber AN, Tauszig-Delamasure S, Hoffmann JA, Lelievre E, Gascan H et al (2003). Nat Immunol 4, 794–800. 10.1038/ni955 12872120

[12] Rutschmann S, Jung AC, Hetru C, Reichhart JM, Hoffmann JA, Ferrandon D. (2000) The Rel protein DIF mediates the antifungal but not the antibacterial host defense in Drosophila. Immunity 12, 569–80. 10843389 10.1016/s1074-7613(00)80208-3

[13] Erturk-Hasdemir D., Broemer M., Leulier F., Lane W. S., Paquette N., Hwang D., Kim C. H., Stoven S., Meier P., and Silverman N. (2009) Two roles for the Drosophila IKK complex in the activation of Relish and the induction of antimicrobial peptide genes. *Proc Natl Acad Sci U S A* 106, 9779–9784. 10.1073/pnas.0812022106 19497884 PMC2701001

[14] Stoven S, Silverman N, Junell A, Hedengren-Olcott M, Erturk D, Engstrom Y, Maniatis T, Hultmark D. (2003). Caspase-mediated processing of the Drosophila NF-kappaB factor Relish. Proc Natl Acad Sci U S A. 100, 5991–6. 10.1073/pnas.1035902100 12732719 PMC156314

[15] De Gregorio E, Spellman PT, Tzou P, Rubin GM, Lemaitre B. (2002) The Toll and Imd pathways are the major regulators of the immune response in Drosophila. EMBO J. 21, 2568–79. 10.1093/emboj/21.11.2568 12032070 PMC126042

[16] Myllymäki H, Rämet M. JAK/STAT pathway in Drosophila immunity (2014). Scand J Immunol 79, 377–85. 10.1111/sji.12170 24673174

[17] Micchelli C. A. & Perrimon N. 2006 Evidence that stem cells reside in the adult *Drosophila* midgut epithelium. *Nature*, 439, 475–9. 10.1038/nature04371 16340959

[18] Ohlstein B. & Spradling A. 2006 The adult *Drosophila* posterior midgut is maintained by pluripotent stem cells. *Nature*, 439, 470–4. 10.1038/nature04333 16340960

[19] Buchon N., Broderick N. A., Chakrabarti S. & Lemaitre B. (2009). Invasive and indigenous microbiota impact intestinal stem cell activity through multiple pathways in *Drosophila*. *Genes Dev*, 23, 2333–44. 10.1101/gad.1827009 19797770 PMC2758745

[20] Amcheslavsky A., Ito N., Jiang J. & IP Y. T. (2011). Tuberous sclerosis complex and Myc coordinate the growth and division of *Drosophila* intestinal stem cells. *J Cell Biol*, 193, 695–710. 10.1083/jcb.201103018 21555458 PMC3166862

[21] Boulanger N, Ehret-Sabatier L, Brun R, Zachary D, Bulet P, et al (2001) Immune response of *Drosophila melanogaster* to infection with the flagellate parasite *Crithidia* spp. Insect Biochemistry and Molecular Biology 31: 129–137. 11164335 10.1016/s0965-1748(00)00096-5

[22] Wilfert L, Longdon B, Ferreira AGA, Bayer F and Jiggins FM (2011) Trypanosomatids are common and diverse parasites of *Drosophila*. Parasitology 138, 858–865. 10.1017/S0031182011000485 21554843

[23] Rowton ED, McGhee RB (1978) Population dynamics of *Herpetomonas ampelophilae*, with a Note on the systematic of *Herpetomonas* from *Drosophila spp*. Journal of Protozoology 25: 232–235.

[24] Rowton ED, McGhee RB (1983) Transmission of Herpetomonas in Laboratory Populations of Drosophila melanogaster. Journal of Protozoology 30: 669–671.

[25] Hamilton PT, Votýpka J, Dostálová A, Yurchenko V, Bird NH, Lukeš J, Lemaitre B, Perlman SJ. (2015). Infection Dynamics and Immune Response in a Newly Described Drosophila-Trypanosomatid Association. MBio. 6, e01356–15. 10.1128/mBio.01356-15 26374124 PMC4600116

[26] Hughes AL and Piontkivska H (2003) Phylogeny of Trypanosomidae and Bodonidae (Kinetoplastida) based on 18s rRNA: Evidence of paraphyly of Trypanosoma and six other genera Mol Biol Evol 20, 644–652 10.1093/molbev/msg062 12679543

[27] Borghesan TC, Ferreira RC, Takata CS, Campaner M, Borda CC, Paiva F, Milder RV, Teixeira MM, Camargo EP (2013). Molecular phylogenetic redefinition of Herpetomonas (Kinetoplastea, Trypanosomatidae), a genus of insect parasites associated with flies. Protist 164, 129–52. 10.1016/j.protis.2012.06.001 22938923

[28] Ebbert MA, Marlowe JL, Burkholder JJ. (2003) Protozoan and intracellular fungal gut endosymbionts in *Drosophila*: prevalence and fitness effects of single and dual infections. J Invertebr Pathol. 83: 37–45 12725810 10.1016/s0022-2011(03)00033-8

[29] Hurd H Host fecundity reduction: a strategy for damage limitation? (2001). Trends Parasitol. 17, 363–8.26. 11685895 10.1016/s1471-4922(01)01927-4

[30] Batistoti M1, Cavazzana M Jr, Serrano MG, Ogatta SF, Baccan GC, Jankevicius JV, Teixeira MM, Jankevicius SI. Genetic variability of trypanosomatids isolated from phytophagous hemiptera defined by morphological, biochemical, and molecular taxonomic markers (2001). J Parasitol. 87 1335–41. 10.1645/0022-3395(2001)087[1335:GVOTIF]2.0.CO;2 11780818

[31] Suster ML, Seugneut L, Bate M, Sokolowski (2004). Refining GAL4-driven transgene expression in *Drosophila* with a GAL80 enhancer trap. Genesis 39, 240–245. 10.1002/gene.20051 15286996

[32] Ha EM, Oh CT, Bae YS and Lee WJ (2005). A direct role for dual oxidase in Drosophila gut immunity. Science 310, 847–510 10.1126/science.1117311 16272120

[33] Ryu JH, Kim SH, Lee HY, Bai JY, Nam YD et al (2008). Innate immune homeostasis by the homeobox gene caudal and commensal-gut mutualism in *Drosophila* Science 319, 777–782 10.1126/science.1149357 18218863

[34] Cronin S. J., Nehme N. T., Limmer S., Liegois S., Pospisilik J. A., Schramek D., Leibbrandt A., Simoes et al (2009). Genome-wide RNAi screen identifies genes involved in intestinal pathogenic bacterial infection. *Science*, 325, 340–3. 10.1126/science.1173164 19520911 PMC2975362

[35] Wiklund ML1, Steinert S, Junell A, Hultmark D, Stöven S. (2009). The N-terminal half of the Drosophila Rel/NF-kappaB factor Relish, REL-68, constitutively activates transcription of specific Relish target genes. Dev Comp Immunol 33, 690–6. 10.1016/j.dci.2008.12.002 19135474

[36] Bach EA, Ekas LA, Ayala-Camargo A, Flaherty MS, Lee H, Perrimon N, Baeg GH (2007) GFP reporters detect the activation of the Drosophila JAK/STAT pathway in vivo. Gene Expr Patterns 7, 323–31. 10.1016/j.modgep.2006.08.003 17008134

[37] Wang Z, Hang S, Purdy A, and Watnick P.I. (2013) Mutations in the IMD pathway and Mustard counter *Vibrio cholera* suppression of intestinal stem cell division in *Drosophila*. (2013) Mbio 8, 00337–13 10.1128/mBio.00337-13 PMC3684835 23781070

[38] Chakrabarti S., Liehl P., Buchon N. & Lemaiter B. (2012). Infection-induced host translational blockage inhibits immune responses and epithelial renewal in the *Drosophila* gut. *Cell Host Microbe*, 12, 60–70. 10.1016/j.chom.2012.06.001 22817988

[39] Hu C. and Aksoy S. (2006). Innate immune responses regulate trypanosome parasite infection of the tsetse fly *Glossina morsitans morsitans*. Mol. Microbiol 60, 1194–1204. 10.1111/j.1365-2958.2006.05180.x 16689795

[40] McName S and Dytham C (1993) Morphometric discrimination of the sibling species *Drosophila melanogaster* (Meigen) and *D*. *simulans* (Sturtevant) (Diptera: Drosophilidae) Syst Ent 18, 231–236.

[41] Singh SR, Mishra MK, Kango-Singh M and Hou SX (2012). Generation and staining of intestinal stem cell lineage in adult midgut Singh SR (ed.), Somatic Stem Cells: Methods and Protocols, Methods in Molecular Biology, vol. 879 10.1007/978-1-815-4_4 Springer Science Business Media LLC 2012. PMC7461621 22610553

