# Supplementary figures and images for: Intestinal NF-κB and STAT signalling is important for uptake and clearance in a Drosophila-Herpetomonas interaction model
Source: PLoS Genet. 2019 Mar 1;15(3):e1007931. doi: 10.1371/journal.pgen.1007931 (PMC6415867; doi:10.1371/journal.pgen.1007931)

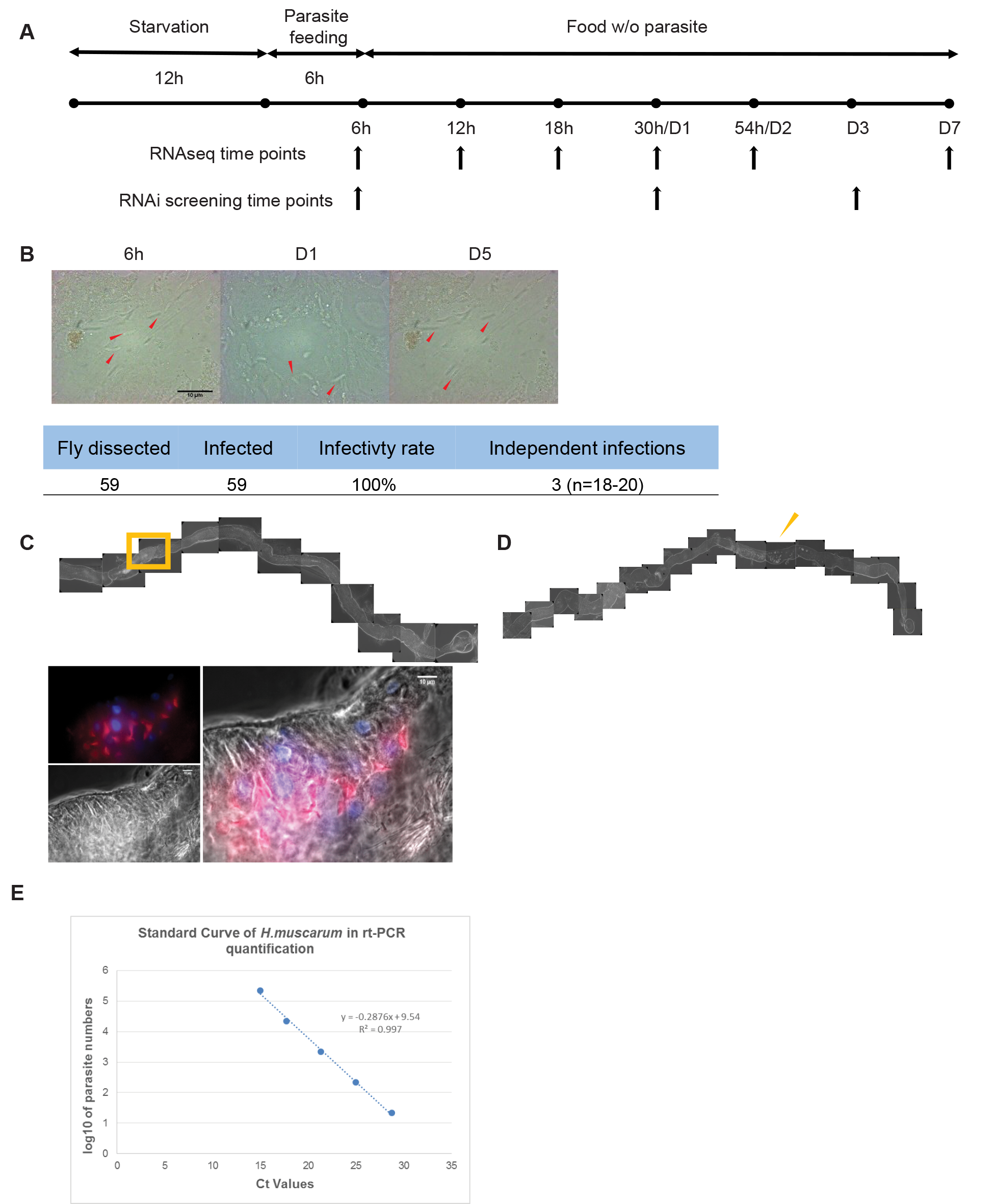

Supplement: S1 Fig — (A) Infection protocol showing the time points for both RNA-seq as well as sampling for parasite numbers following various RNAi treatments. (B) Parasite infection was also verified by studying intestines manually at different time points. (C) Pre-stained parasites with DAPI (blue) and mitotracker (red) was seen in the anterior (C) and posterior midgut (D), where close-ups (from C) indicated the formation of rosettes reminiscent of Leishmania. (E) A representative standard curve that was made each time with the parasite culture used to infect, so as to help quantify absolute numbers of parasites in infection experiments. (TIF) [file pgen.1007931.s001.tif]

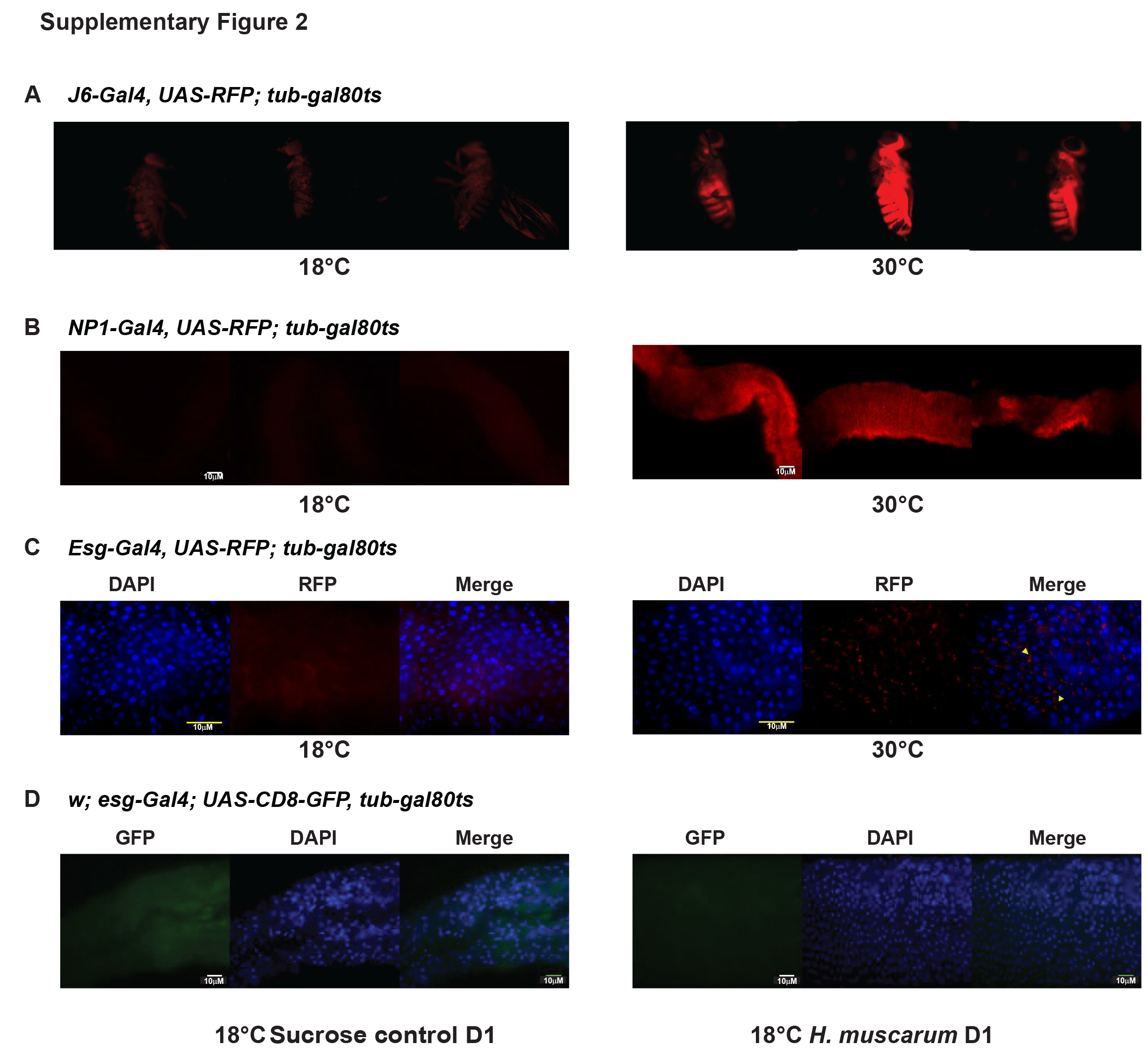

Supplement: S2 Fig — (A) Using UAS-RFP to investigate the control of GAL80ts over the GAL4 drivers used in this study namely, the general driver J6-GAL4 (B) the EC-specific driver NP1-GAL4 and (C) the ISC/EB-specific Esg-GAL4. In all of these GAL4-GAL80ts combinations RFP was only induced at 30°C. (D) At the restrictive temperature (18°C) the system was not inducible following infection. (TIF) [file pgen.1007931.s002.tif]
